# Neurological Soft Signs (NSS) in Census-Based, Decade-Adjusted Healthy Adults, 20 to >70 Years of Age

**DOI:** 10.3389/fpsyt.2021.670539

**Published:** 2021-06-24

**Authors:** Silke Bachmann, Michaela Beck, Dai-Hua Tsai, Friederike Haupt

**Affiliations:** ^1^Department of Psychiatry, University Hospitals of Geneva, Geneva, Switzerland; ^2^Department of Psychiatry, University Hospitals and Martin-Luther University, Halle, Germany; ^3^Geriatriezentrum Zwenkau, Sana Kliniken AG, Zwenkau, Germany; ^4^Swiss Centre for Occupational and Environmental Health (SCOEH), Winterthur, Switzerland; ^5^Private Practice, Halle, Germany

**Keywords:** neurological soft signs, healthy adults, census-based, decade adjusted, age 20–70+, increase

## Abstract

Neurological soft signs (NSS) represent minor neurological features and have been widely studied in psychiatric disease. The assessment is easily performed. Quantity and quality may provide useful information concerning the disease course. Mostly, NSS scores differ significantly between patients and controls. However, literature does not give reference values. In this pilot study, we recruited 120 healthy women and men to build a cross-sectional, census-based sample of healthy individuals, aged 20 to >70 years, subdivided in 10-year blocks for a close approach to the human lifeline. Testing for NSS and neurocognitive functioning was performed following the exclusion of mental and severe physical illness. NSS scores increased significantly between ages 50+ and 60+, which was primarily accountable to motor signs. Gender and cognitive functioning were not related to changes of scores. Although the number of individuals is small, study results may lay a foundation for further validation of NSS in healthy individuals.

## Introduction

The term neurological soft signs (NSS) refers to subtle and distinctive neurological abnormalities which are not as obvious as hard, i.e., localized, pathology, such as nuclear or primary tract lesion (1–3). Nevertheless, they reflect deficits in sensory integration, motor coordination, sequencing of complex motor acts, and others. Thus, and in combination with the relatively effortless assessment, NSS have been established in psychiatry as a meaningful tool to differentiate for example disease entities, course types, and degrees of chronicity. Recently, the motor domain of NSS in psychiatric illness in general has attracted increasing attention (4–8).

### NSS in Psychiatric Patients

Psychiatrists and researchers have been interested in NSS at least for the past four decades (9). The highest scores of NSS were found in schizophrenia patients (10), both quantitatively and qualitatively (11). NSS in the pathological range were also detected in a variety of patients with—among others—mood (12), obsessive–compulsive (13), and personality disorders (14). Nevertheless, individuals who suffer from schizophrenia have been studied most often. Researchers found that NSS are rather not related to medication (10, 15–19), since they are present in first-episode patients (10, 20–28) as well as in individuals at risk and in relatives (29–33). Moreover, NSS are associated with negative symptoms, formal thought disorders, and cognitive symptoms in general (4, 17, 26, 34–41), as well as with fluctuations of psychopathology over time (20, 25, 26, 42, 43).

### NSS in Healthy Individuals

It is well-established that NSS can be found in healthy subjects as well, numbers range from 0 to 54% (11, 16, 44–55). This is an unusually wide range suggesting a significant heterogeneity regarding methodological issues at the level of defining and assessing NSS but also at the level of study sample recruitment and doubtlessly the presence of NSS. Concerning recruitment, information often is missing with respect to paternal age, malnutrition or infections during pregnancy, complications during pregnancy and delivery, and the complete family history, i.e., presence of psychiatric disease—all of which constitute risk or causal factors for the development of NSS.

NSS are not dichotomously distributed, but their presence seems to depict a continuum between healthy individuals and psychiatric patients. This has been shown by studies on schizophrenia patients' relatives, whose scores take an intermediate position between healthy and schizophrenia subjects, e.g. (31), again depending on quantity (number of signs) and quality (markedness) of the signs. Along these lines, in a systematic review and meta-analysis, Neelam and Marshall (56) confirmed that schizophrenia patients scored higher on NSS than relatives whose scores again ranged higher than those of unrelated healthy controls.

However, evidence concerning the presence of NSS in healthy individuals is mostly based on the assessment of matched control subjects in studies on psychiatric patients, i.e., healthy subjects have hardly been looked at independently and systematically in different age groups, according to gender, and according to stability over time, while approximations might be drawn from studies such as that of Chan et al. (57).

In the present study, we thus addressed the old but still prevalent critique that instruments for NSS assessment lack normative data (58) by presenting a preliminary set of NSS scores from an adult population sample applying the Heidelberg NSS scale (H-NSS) (59, 60) to different age groups. These data may provide a first step toward the establishment of NSS norm values.

### Neuronal Networks

Apart from providing a useful tool in clinical contexts, NSS have added up to modeling cerebral networks involved in schizophrenia. The dysfunctional networks and related brain areas associated with the pathogenesis of NSS have not been fully identified yet. Neuroimaging studies suggest a link to activation changes in the sensorimotor cortex and the supplementary motor area, cerebellar abnormalities, and subcortical findings involving the basal ganglia and thalamus (22, 27, 28, 35, 61–63). These findings are substantiated by the “cognitive dysmetria” hypothesis by Andreasen, based on the so-called cortico-cerebellar-thalamic-cortical circuitry (64). The latter has widely been used to explain the manifold symptoms of schizophrenia and to integrate motor and cognitive symptoms.

### Purpose and Hypotheses

This study set out to establish reference values of a representative, cross-sectional, census-based sample of healthy individuals, ages 20 to >70 years, subdivided in 10-year blocks for a close approach to the human lifeline. A longitudinal study would have been the most adequate design, but our resources did not allow following a large group over four or five decades. Children and teenagers were excluded because they naturally exhibit higher NSS scores due to ongoing brain maturation which comes to a standstill around the age of 20, when they mostly disappear and reach their best, i.e., lowest, values (65, 66). Brain changes with increasing age have been described and concern, e.g., whole-brain volume (WBV), white matter (WM) integrity, metabolism, and cognitive functioning (67–70). We thus hypothesized that NSS become more prominent with age, in terms of quantity and quality, and in line with the normal aging processes. Neurocognitive tests were included using tests with high-quality criteria and age-related norms to (a) provide a solid matrix against which to contrast NSS results and to (b) compare information on brain circuits provided by both approaches and to possibly obtain a clearer picture. Moreover, correlations between NSS and cognitive functioning are well-established (11, 71–73).

## Materials and Methods

### Subjects

During childhood and adolescence, NSS represent correlates of the developing brain. Probands thus had to be 20 years of age or older. Men and women, separately, from the normal population in Halle (Saale) were recruited via advertisements. Selection was performed according to the German census with respect to gender and education. A fixed-ratio schedule was used, including six age-specific groups (20–29, 30–39, 40–49, 50–59, 60–69, and >70 years). Age being the variable of interest, no age limit was set for the higher age groups. Representing the differences in life expectancy, 11 women and 9 men were recruited into the group >70 years, whereas equal numbers of men and women were included into all other groups. Probands' educational status was matched to the respective decade of the general German population according to the census data of 2008 (74). Recruitment was performed during the years 2009 and 2010.

Originally, we had aimed at including 180 adults (75). The intended cell size of 15 individuals per age decade and gender was not reached due to recruitment problems. The size had to be narrowed to *n* = 10 per cell. Likewise, the intended sample size had to be narrowed from 180 to 120 individuals.

### Inclusion Criteria

Subjects had to be healthy and 20 years or older without upper age limit because age was the independent variable. The Structured Clinical Interview for DSM-IV Axis I (SCID-I, German version) (76) screening questionnaire was used to establish mental health; physical health and medication were assessed by a semi-structured interview. Exclusion criteria were any current or former psychiatric or severe somatic illness, any medication which acts on the central nervous system, refusal to participate in the study, or withdrawal of consent.

### Instruments

All probands underwent semi-structured interviewing to exclude lifetime serious physical, especially neurological, disease, regular intake of psychopharmacological medication, and psychiatric disorders by applying the SCID screening interview.

NSS were assessed with the Heidelberg NSS scale (H-NSS) (59, 60), which consists of 16 items, most of which (namely, 11 items) are rated separately for both the right and left sides, which is not the case for the items gait, tandem gait, Ozeretzki's test, articulation, and right/left orientation. The items are grouped into five factor subscales: motor coordination (items: Ozeretzki's test, diadochokinesis, pronation/supination, finger–thumb opposition, and articulation); sensory integration (items: gait, tandem gait, and 2-point discrimination); complex motor signs (items: finger-to-nose-test, fist–edge–palm test); right–left and spatial orientation (items: right/left orientation, graphesthesia, face–hand sensory test, and stereognosis); and hard signs (items: arm-holding test and mirror movements). Each single soft sign is rated on a 0–3-point scale (no/slight/moderate/marked abnormality, respectively), leading to scores between 0 (minimum) and 81 (maximum). On construction, the H-NSS yielded good internal reliability (Cronbach's alpha = 0.89 for healthy subjects) as well as interrater reliability (*r* = 0.88, *p* < 0.005) (59). Prior to this study, two raters (F.H. and M.B.) were trained together until they reached high interrater reliability. The training was performed by an experienced rater (S.B.).

The NSS assessment was complemented by an establishment of handedness via the Edinburgh Inventory (77). A laterality quotient was calculated that ranges from −100 (strong left hander) to +100 (strong right hander) from the rating of 10 common activities (e.g., please demonstrate how you throw a ball). We applied narrow definitions of right-handedness (laterality quotient of +80) and left-handedness (laterality quotient −80 or less); the remaining range represented mixed-handedness. Complementary questions concerned regular practice of music and left-handers in the family.

Intelligence was measured by applying the “Mehrfachwahl-Wortschatz-Test” (MWT-B; a German language-based verbal intelligence test) (78), which assesses the general, crystallized intelligence quotient (IQ) by testing recognition of vocabulary (37 items of increasing difficulty).

### Neurocognitive Testing

Well-known neurocognitive tests with good psychometric properties were administered; see Table 1.

**Table 1:** Demographic characteristics of healthy subjects (*n* = 120), descriptive data, in count or in median (Q1–Q3).

**Age groups^a^**	**20–29**	**30–39**	**40–49**	**50–59**	**60–69**	**70+**	**Overall**
**Education level^b^**							
Minimal level or no certificate	1	0	0	2	5	7	15
Average level certificate	11	13	14	15	13	10	76
High level certificate	8	7	6	3	2	3	29
**IQ**	107 (101–112)	105.5 (104–124)	112 (101.8–128.5)	121 (112–130)	105.5 (100–128.5)	124 (113.5–130)	112 (104–124)
**Verbal fluency test**
Words beginning with S	15.5 (11.5–20)	13 (11–15)	18 (12–21.5)	16 (12–18)	13 (10–14)	13 (9–17)	14 (11–18)
Words naming flower or sport	17 (14.5–18)	16 (13–17.5)	18 (15–20)	16 (14.5–17)	14 (13–17)	14 (12–16)	16 (14–18)
**Complex Figure Test (CFT)**
CFT 1: copy	35 (33–36)	35 (33–36)	35.5 (34.5–36)	34.5 (33.5–35)	34 (31–36)	34 (31–35)	35 (33–36)
CFT 2: recall	26 (21–28.5)	22.5 (18–27)	19 (16.8–24)	20.5 (15.3–23)	15.5 (10–20)	17 (13–19.5)	19.8 (15–25)
**Concentration test (d2)**
Corrected for negatives/false positives	175.5 (154.5–205.5)	168 (147.5–190)	155 (126.5–182.5)	146.5 (128.5–169)	132 (101–150)	119.5 (101–143)	150 (128–179)
**Logical Memory (LM)**
Immediate recall (LM I)	29.5 (27.5–32.5)	27.5 (24–32.5)	31 (25.5–33)	26.5 (24–29.5)	23 (20.5–28)	24 (18–27)	27 (23–31)
Delayed recall (LM II)	26.5 (22–30.5)	25 (20.5–28)	27.5 (18–33)	20 (17.5–26)	20 (14.5–27.5)	20 (16–23)	22 (19–28)
**Digit span memory**
Forward	8 (6.5–10)	7 (6–8.5)	8 (6.5–10)	8 (6.5–9)	8 (5–9)	7 (6–8)	7 (6–9)
Backward	6.5 (5–8)	6 (5–8)	6.5 (6–8)	6 (5–7)	6 (5–6)	5 (4–6)	6 (5–7)
**Trail Making Test (TMT)**
TMT A: alphabet	21 (19.3–24)	20.5 (17.5–24)	22 (19–29)	25.5 (20.5–31)	35 (25–41)	42 (37–53)	25 (21–37)
TMT B: alternating alphabet: numbers	47 (40–59.2)	57 (45.5–66.5)	48 (40–67.5)	61 (50.5–80)	79 (60–96)	95 (81–115)	61 (47–84)

a *50% men and 50% women in each age group except for the group 70+ according to census*.

b *Education level matched to German census for each age group*.

The Rey Osterieth complex figure test (CFT) (79), according to Strauss et al. (80), asks probands to copy a complex figure (copy trial, CFT I) and to redraw this figure from memory after about 30 min (delayed recall, CFT II). Hereby, memory (encoding, storage, and retrieval) as well as visuospatial abilities (recall, recognition, and construction) are being tested. CFT results are most likely linked to the cerebellum and its functioning (81).

The tests “Logisches Gedächtnis I/II” (LG, logical memory) are subtests taken from the German adaptation of the Wechsler Memory Scale, revised version (WMS-R) (82). Both subtests are based on two stories which are read to the proband once (twice in older subjects). Logical, episodic, and long-term memories are assessed via immediate free reproduction of the stories (immediate recall, logical memory I) as well as free reproduction after about 30 min (delayed recall, logical memory II), the latter being followed by complementary, closed questions concerning details (yes/no; recognition).

The digit span memory test is also taken from the WMS-R. Two to nine numbers have to be repeated either forward or in reverse order. According to PET studies (83, 84), both the logical memory (LG) test and the symbol digit modalities test (DSMT) (85), which partially resembles the digit span memory test, give information regarding prefrontal and cerebellar functioning.

The d2 test depicts concentration in terms of selective and sustained attention with visual search function and processing speed influencing the result (86). Several rows of “d” and “p” with one to two lines below and/or one to two lines above the letter are presented. The proband is instructed to mark all “d”s with two lines. Concentration ability is calculated from correct and false-positive as well as true-negative answers.

Trail-Making Tests A and B (TMT-A and TMT-B, respectively) (87) measure visual search function, attentiveness, speed, and mental flexibility. Whereas, TMT-A asks to link numbers, TMT-B requests to shift between numbers and letters. Both subtests represent a measure of frontal activity.

Verbal fluency was studied via the most commonly used subtests of the German “Regensburger Wortflüssigkeitstest” (RWT) (88). Probands are asked to name as many words as possible which start with an S, followed by naming words which start with an M in a given time (formal lexicon), as many animals and as many sports and plants while switching between the latter (semantics). Hereby, divergent thinking and cognitive flexibility are assessed, which are associated with frontal areas. For feasibility reasons, we limited testing to the S-words and to the switch between sports and plants.

In sum, general explanations, written informed consent, assessment of demographic data and health status, and testing time amounted to a total of 90–100 min per person. Resting periods were allowed according to individual needs. The study was performed in accordance with the Declaration of Helsinki (89).

### Statistics

According to our hypothesis, NSS become more prominent with age, in terms of both quantity and quality; age was defined as the independent variable, and NSS total score as the dependent variable. Gender, educational level, and IQ were confounding factors. Linear regression models were used to test for associations between NSS total score and age by adjusting for gender and IQ.

Differences in NSS scores with respect to age groups were detected with Kruskal–Wallis tests, and if present *post-hoc* Dunn's tests were carried out to identify which groups differed from one another. Spearman correlations were performed for NSS total score vs. IQ, handedness, and the results of cognitive testing.

The initial study recruitment aimed at including 180 adults, in order to estimate errors for six age groups (from 20+ to 70+, with a 10-year increase per group). The current study used age as a continuous variable. Browne has mentioned a general flat rule to “use at least 30 subjects or greater to estimate a parameter” (90). The sample size was 120 subjects, indicating sufficient power to investigate the association between age and NSS total score (90). In order to avoid type 1 error, significance level was chosen to be 1% (0.01). All statistical analyses were conducted using STATA Special Edition 15.0 (91).

## Results

The presented sample was homogeneous and census based, consisting of white Germans from one area. Descriptive data are shown in Tables 1, 2. All participants took part in the interviews and the assessments of NSS and handedness. One person in the 60+ group did not take part in all neurocognitive tests, but in IQ and memory testing (LG I and II) only. In the 70+ group, one individual was not able to perform any of the cognitive tests, whereas another omitted to take the d2 concentration test.

**Table 2:** Distribution of NSS total and each single NSS in each age group, descriptive data; median (Q1–Q3); *r*, right, *l*, left.

**Age groups**	**20–29**	**30–39**	**40–49**	**50–59**	**60–69**	**70+**
NSS total	7 (4–8.5)	5.5 (4–6.5)	6.5 (4.5–10)	8 (7–10.5)	16 (12–17)	15 (12.5–7.5)
NSS 1	0	0	0	0	0	0 (0–1)
NSS 2	0 (0–0.5)	0 (0–1)	0 (0–1)	1 (0–1)	1 (0–1)	1 (1–2)
NSS 3	0 (0–1)	0 (0–1)	0 (0–1)	0 (0–1)	0 (0–1)	0 (0–1)
NSS 4	0.5 (0–1)	0 (0–1)	0 (0–1)	0 (0–1)	0 (0–1)	1 (0–1)
NSS 5	0 (0–0.5)	0 (0–0.5)	0.5 (0–1)	0.5 (0–1)	1 (1–2)	1.5 (1–2)
NSS 6r	0	0	0	0	0	0 (0–1)
NSS 6l	0	0	0	0 (0–1)	0	0 (0–1)
NSS 7r	0 (0–0.5)	0 (0–0.5)	0 (0–1)	1 (0–1)	0.5 (0–1)	1 (0–1)
NSS 7l	0	0	0 (0–1)	0 (0–1)	0.5 (0–1)	1 (0–1)
NSS 8r	0	0	0	0	0 (0–1)	0 (0–1)
NSS 8l	0	0	0 (0–1)	0	0 (0–1)	0 (0–1)
NSS 9r	0	0	0	0	0	0 (0–1)
NSS 9l	0	0 (0–0.5)	0 (0–0.5)	0 (0–0.5)	0 (0–1)	1 (0–1)
NSS 10r	0 (0–1)	0	0 (0–0.5)	0 (0–1)	1 (0.5–2)	1 (0–1)
NSS 10l	0 (0–0.5)	0 (0–1)	0 (0–0.5)	0 (0–1)	1 (0.5–1)	1 (0–1)
NSS 10r	1 (1–1)	0 (0–1)	0.5 (0–1)	1 (0.5–2)	0.5 (0–1)	1 (1–1)
NSS 11l	1 (0–2)	0.5 (0–1)	1 (0–1)	1 (0–2)	1 (0–2)	1 (1–1)
NSS 12r	0	0	0	0	0.5 (0–1)	0 (0–1)
NSS 12l	0	0	0	0	0 (0–1)	0.5 (0–1)
NSS 13r	0	0 (0–1)	0	0	0 (0–1)	1 (0–1)
NSS 13l	0 (0–0.5)	0 (0–1)	0 (0–1)	0 (0–1)	0.5 (0–1)	0 (0–0.5)
NSS 14r	0	0	0	0	0 (0–1)	0 (0–1)
NSS 14l	0	0	0	0	0	0
NSS 15r	0	0	0	0	0 (0–1)	0 (0–1)
NSS 15l	0	0	0	0 (0–0.5)	0 (0–1)	0 (0–0.5)
NSS 16r	0 (0–0.5)	0	0 (0–0.5)	0 (0–1)	1 (1–1)	1 (0–1)
NSS 16l	0	0	0	0	1 (0.5–1)	0.5 (0–1)

According to the census, gender was not equally distributed in the age group 70+. However, none of the assessed variables differed between men and women. Also, according to the census data, levels of schooling did not differ between the sexes. One single person in the age group 20+ remained without a formal certificate and was incorporated into the next higher group. Also, one female subject had not reached her 20th birthday yet. She was included due to recruiting problems within the given time. Education, IQ, results of cognitive testing as well as handedness, and NSS were comparable between men and women.

Although age groups differed regarding IQ, no influence of IQ on NSS emerged. The type of school attended was not related to IQ because there was a shift toward higher education from a maximum of 10% in a given age group in the 60s and 70s to over 50% attendance of the school type preparing for university studies in 2015 (East and West Germany alike).

### NSS

**Figure 1:**
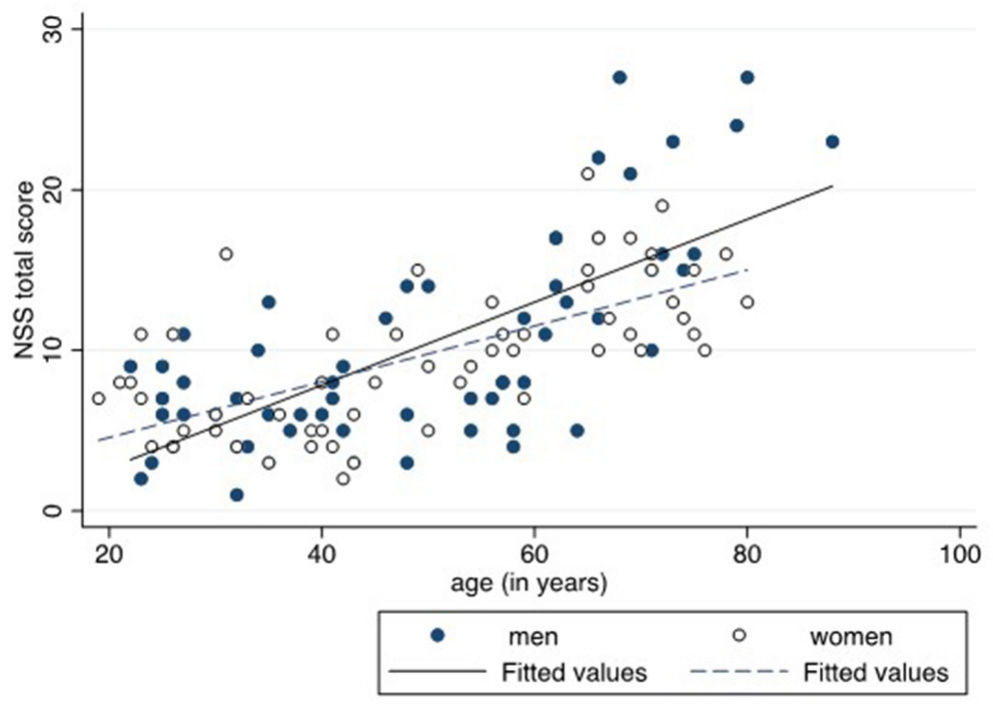
Relationship between neurological soft signs (NSS) total score and age by sex.

NSS were overall in the normal, i.e., physiological range up to the age of 50+. Age groups 60+ and 70+ exhibited scores which compare to minor abnormalities (scores 9–15) and thus to the lowest level of possible abnormalities (scores 9–81) (92, 93). Due to the high amount of individuals scoring in the physiological range, the overall distribution of NSS was not normal but skewed to the left. On the whole, the NSS total scores were significantly related to age in both men and women, as shown by the regression curves in Figure 1, the curve being slightly steeper in men. For the whole sample, with a 10-year increase in subjects' age, the NSS total score rose by 2.4 points (coefficient 0.24, 95% confidence interval 0.21–0.28, *p* < 0.001) after adjusting for gender and IQ. For men, with a 10-year increase in subjects' age, the NSS total score rose by 2.6 points (coefficient 0.26, 95% confidence interval 0.20–0.33, *p* < 0.001) while the increase for women was 2.0 points (coefficient 0.20, 95% confidence interval 0.15–0.26, *p* < 0.001), both after adjusting for IQ.

**Figure 2:**
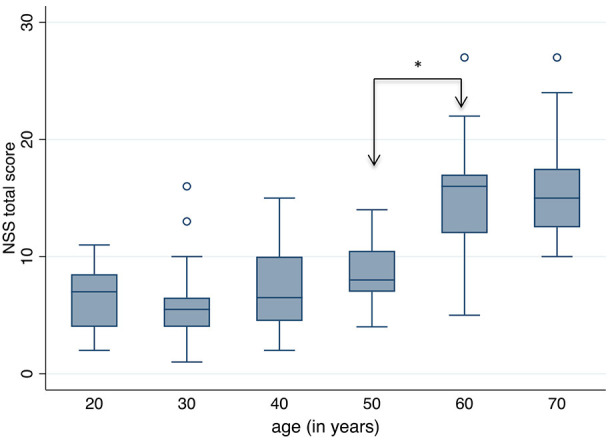
Neurological Soft Signs (NSS) total score according to age groups. A significant increase, i.e. worsening of NSS, is seen between the ages 50 and 60 years (*Dunn's Pairwise Comparison, *p* = 0.0002).

The box plots of the NSS total score in each age group is shown in Figure 2. The most significant increase, i.e., worsening of NSS, is seen between the age groups 50+ and 60+. The following single signs accounted for this increase: gait, tandem gait, Ozeretzki's test, finger–thumb opposition, 2-point discrimination, fist–edge–palm test (NSS 1, 2, 5,10, 12, and 16 in this order, both sides for signs 10, 12, and 16; see Supplementary Tables 2–10). These discriminating signs refer to motor tasks, i.e., either to complex motor tasks (fist–edge–palm test) or to motor coordination (Ozeretzki's test and finger-to-thumb test) and to sensory integration (gait, tandem gait, and 2-point discrimination). The remaining tests carry less weight toward the NSS increase between ages 50+ and 60+.

Handedness scores ranged from −40 to +100. The majority of subjects, namely, 90.9%, was right-handed; 11 subjects were mixed-handed; left-handedness did not occur. Presence of left-handers in the family (one to nine per age group) or regular practice of a musical instrument during the past 6 months (zero to four per age group) were unrelated to handedness. Handedness itself was not associated with any of the other parameters; neither were NSS scores or results of cognitive tests.

### Neurocognitive Testing

Neurocognitive test results of individuals in this study (see Table 3) were compared to the respective age groups' norms as given in the test manuals with one exception: probands' decrease of concentration in the sixth and more so the seventh decades could not be compared to norm values because the d2 test standardization exists up to the age of 60 years only.

**Table 3:** Spearman correlation between co-function and total NSS.

	**VTF s**	**VTF change**	**CFT 1**	**CFT 2**	**d2**	**LG I**	**LG II**	**DSM forward**	**DSM backward**	**TMT-A**	**TMT-B**	**NSS total score**
VFTs	1.00											
VFT change	0.50^*^	1.00										
CFT 1	0.20	0.28^*^	1.00									
CFT 2	0.23	0.30^*^	0.49^*^	1.00								
d2	0.41^*^	0.47^*^	0.41^*^	0.44^*^	1.00							
LG I	0.28^*^	0.35^*^	0.16	0.27^*^	0.43^*^	1.00						
LG II	0.33^*^	0.36^*^	0.17	0.34^*^	0.45^*^	0.81^*^	1.00					
DSM forward	0.24^*^	0.38^*^	0.34^*^	0.25^*^	0.34^*^	0.14	0.09	1.00				
DSM backward	0.29^*^	0.34^*^	0.38^*^	0.45^*^	0.45^*^	0.22	0.22	0.50^*^	1.00			
TMT-A	−0.34^*^	−0.48^*^	−0.30^*^	−0.45^*^	−0.64^*^	−0.28^*^	−0.31^*^	−0.24^*^	−0.49^*^	1.00		
TMT-B	−0.40^*^	−0.48^*^	−0.45^*^	−0.49^*^	−0.71^*^	−0.48^*^	−0.49^*^	−0.27^*^	−0.53^*^	0.77^*^	1.00	
NSS total score	−0.39^*^	−0.43^*^	−0.34^*^	−0.40^*^	−0.61^*^	−0.43^*^	−0.41^*^	−0.25^*^	−0.46^*^	0.57^*^	0.60^*^	1.00

* *p-value < 0.01; whereas higher values refer to better performance in the majority of tests, this is not the case for TMT-A, TMT-B, and NSS*.

*LM I, Logical Memory, immediate recall; VFT s, Verbal Fluency Test, words beginning with S; LM II, Logical Memory test, delayed recall; VFT change, words naming flowers and sports, changing between the two; DSM forward, Digit Span Memory, forwards; CFT1, Complex Figure Test, copy; DSM backward, Digit Span Memory, backwards; CFT2, Complex Figure Test, recall; TMT-A, Trail Making Test, alphabet; d2, Concentration Test, corrected for negatives/false positives; TMT-B, Trail Making Test, alternate alphabet/numbers*.

Two moderate and thus meaningful correlations arose between NSS total score and results from cognitive testing, namely, a negative correlation between NSS total score and concentration according to d2 (*r* = −0.61, *p* < 0.01) as well as a positive correlation between NSS total score and the TMT-A (*r* = 0.57, *p* < 0.01). NSS total score and TMT-B were also positively and significantly correlated (*r* = 0.60, *p* < 0.01).

These correlations point in the same direction, namely, an increase of NSS being related to worsening of concentration and/or visual search function and processing speed with age.

## Discussion

This study aimed to assess NSS in healthy individuals grouped into age decades ranging from 20 to over 70 years, census based with respect to gender and education. The study yields the following main results:

1. It presents a preliminary reference matrix across the adult life span. NSS scores increased with age in this sample of 120 healthy subjects, independently of gender, intelligence, education, and handedness. Not only did NSS increase with age, but there was a period when the most pronounced change occurred, namely, between the fifth and sixth decades of life.
2. Worsening of motor function yielded the most important share of overall NSS increase.
3. The cognitive domains processing speed, visual search function, and concentration worsened in parallel with NSS, but none of the remaining cognitive functions did.

Ad 1: To our knowledge, this is the first study which systematically assessed age-related changes of NSS in healthy subjects on the basis of census data including age and education. Although the sample size is small due to recruitment problems, it encompasses a well-characterized group of adults with similar cell sizes per decade in females and males (20 to >70 years of age).

The NSS total score in the presented sample amounted to 10, which lies above the reference values of healthy subjects' scores as established for the H-NSS scale, namely, 0–8 points (93). However, when looking at younger and older age groups, younger (aged 20–49) individuals' NSS amounted to 6.5 ± 3.3 and thus remained in the physiological range. Reference values for healthy subjects aged 50 and above do not exist. Thus, scores in the latter age group (13.6 ± 5.7) in this study may as well be physiological.

NSS total score increased linearly with age in men and women, the rise being somewhat steeper in men but not significantly so. The increase proved to be independent of IQ, education, and handedness in both sexes. The most obvious and statistically significant increase occurred between the fifth and sixth decades. This is an important result because NSS—being mainly studied in psychiatric patients—have long been looked at as stable (11, 33). Where healthy subjects were studied, they usually served as controls. Even when healthy subjects' NSS were included in longitudinal studies, scores did not change (20), not surprisingly so, because patients were mostly at the beginning of their psychiatric disease, and thus, patients and controls were relatively young. This is especially true for schizophrenia, but also for other mental illness studied, except for the dementias. With ages clearly below 50 years and follow-up periods of 2–10 years at most, matched controls usually did not reach the age when a decrease in NSS might be expected.

There are a few possible exemptions, namely, the groups of Chan, Fountoulakis, and Kodama (49, 57, 94). These researchers sought to shed light on NSS in healthy individuals who were not recruited as controls. Kodama et al. (94) performed gross neurological examinations on healthy subjects aged 60–80 years cross-sectionally. The type of examination, however, is not comparable to the NSS assessment. Moreover, young and middle-aged groups are lacking. Fountoulakis et al. (49) studied 122 healthy subjects to detect a possible presence of NSS. There was no significant correlations between NSS and age, gender, or education. However, their focus was not on age, and although the age span ranged from 18 to 65 years with a mean of 33 years, only 19% of the subjects were above 40 years of age. Thus, most individuals did not reach the critical age where NSS increase began in our study. Chan et al. (57) assessed NSS in a large sample of patients with schizophrenia and other psychiatric disorders, in individuals with schizotypal personality disorders, and in unaffected relatives as well as healthy subjects aged 14–62 (*n* = 1,224). They matched healthy subjects to schizophrenia patients, including gender and intellectual level, which led to a very young sample with 74% of participants aged below 30 (80% below 40) years, whereas we collected probands based on a census, thus reaching an equal distribution between all age groups up to 70+. IQ was not normally spread in both studies, whereas gender was. NSS were rated as present or absent by Chan et al. (57); our assessment comprised four levels. Notwithstanding the differences between the studies, the NSS distribution according to age is stunningly similar. Chan et al. depicted a U-shaped curve with a descending left leg and a rising right leg. The left leg reflects the falling NSS scores in teenagers, the right leg an increase with age. The latter starts rising slightly at age 40 and above and curves above the age of 50. This finding strongly supports our results regarding the NSS increase starting at age 40+ and intensifying at age 50+. With a 10-year increase in subjects' age, NSS total score rises by 2.37 points (coefficient 0.237 × 10 = 2.37), after adjusting for gender and IQ. A decrease of NSS in teenagers (falling left leg) refers to the developmental trajectory (65).

In essence, our findings as well as those by Chan et al. (57, 95) are in line with studies on morphology, such as a review of 56 longitudinal MRI studies (69). These authors studied all available data on WBV changes over the life span by applying a weighted regression. Their analysis revealed relative stability of brain volumes between the ages ~18 and 35. At about 35 years, a steady brain loss of 0.2% per year began, which increased to 0.5% annual loss around the age of 50 years and remained stable at 0.5% after the age of 60 years. We assume that the increased brain volume loss, which comes about at age 50, is reflected functionally by worsening of NSS. Along these lines, a study on brain maturation deserves mention. Coupé et al. (96) assessed WM and gray matter (GM) in a large number of individuals to cover the human life span. They found an inverse U-shaped distribution of WM with the largest extension of WM in midlife—thus mirroring Chan's U-shaped distribution of NSS (lowest scores in midlife) and supporting that functioning is at its best level in this age group.

Our study yielded a slightly but non-significantly more pronounced increase of NSS with age in male as opposed to female participants. This is supported by findings of Driscoll et al. (97), who reported a more pronounced volume loss in men relative to women with increasing age. However, results of Coupé et al. and Hedman et al. (69, 96) predominantly oppose the aforementioned results. Coupé et al. examined data of over 2,000 healthy individuals and detected a more pronounced volume loss in males above the age of 80 years only. Hedman et al. (69) reported no gender differences in volume loss over time in the majority of 56 studies reviewed. Chan et al. (57) as well-concluded that change rates of WBVs were independent of gender.

Ad 2: In our study, worsening of motor function and sensory integration yielded the most important share of overall NSS increase between the ages 50 and 60. The following NSS contributed significantly: gait, tandem gait, Ozeretzki's test, finger–thumb opposition, 2-point discrimination, fist–edge–palm test (NSS 1, 2, 5,10, 12, and 16 in this order, both sides for signs 10, 12, and 16; see Supplementary Tables 2–10). According to the original five-factor model of H-NSS (88), gait, tandem gait, and 2-point discrimination represent “sensory integration”; Ozeretzki's test and finger-thumb-opposition belong to the factor “motor coordination,” and the fist–edge–palm test is a “complex motor task.” There was no relation to handedness for either of these signs. When we compare the classification of signs to that in the literature, there is some slight divergence regarding the fist–edge–palm test. It is also grouped with complex motor acts in the Neurological Evaluation Scale Evaluation Scale (NES) (98), but with the motor coordination tests on other scales, e.g., the Cambridge Neurological Inventory (CNI) (95). In either case, an equal share of motor and sensory signs accounts for the overall NSS increase between 50 and 60 years.

Our findings regarding an important role of motor NSS in healthy subjects are in line with those of Fountoulakis et al. (48), where 54% of healthy subjects exhibited one NSS, 14% two NSS, and 6.5% three NSS—among which motor signs were overrepresented. Chan et al. (57) regrettably did not present NSS on an item level; it thus remains unclear which single items contributed to the rise in total NSS. Dazzan et al. (99) studied the relationship of NSS and brain volumes in healthy subjects, differentiating GM and WM. The group reported motor signs to be mostly normal whereas sensory signs were not; the latter were related to GM reductions (see below). Participants' age ranged from 17 to 53 years, with a mean around 30 years. The absence of motor signs in this young age group compares to our results, where motor signs did not increase before the age of 50. Also, three sensory signs rose in our study, beyond the age 50 or 60 as well. Therefore, differences between studies may be attributed to the younger age in Dazzan et al. (99). The throughout higher sensory scores in the latter study may be reconciled with those reported by Chen et al. (100), who described correlations between motor and sensory signs.

Contrary to the longstanding knowledge on motor and supplementary motor areas in general, neuroimaging literature on motor abnormalities in healthy subjects is scarce. The above-mentioned study by Dazzan et al. (99) represents a landmark study. They grouped motor coordination, motor sequencing, and sensory integration to an NSS subscale called “integrative scale.” Healthy subjects were divided into subgroups according to high and low scores on this scale. High integrative scores were significantly related to GM reduction, namely, to the following GM clusters: anterior cingulate bilaterally, right middle temporal gyrus into the inferior frontal gyrus, and the right superior temporal gyrus. Regarding WM, the high-scoring group exhibited reductions of the superior longitudinal fasciculus into the internal and external capsules. Our results are in line with these findings on integrative signs, as sensory and motor signs contributed equally to the overall rise of NSS.

Along these lines (99) and with respect to overall NSS in healthy controls, an association to GM was also reported by Thomann et al. (22). In 2015, Thomann et al. (101) presented a functional imaging study, where in three out of five cortical networks of interest, negative correlations arose between NSS and the right precuneus, right superior frontal areas, supplementary motor areas, and left paracentral gyrus; no association with subcortical areas was detected. Thus, motor control in cortical rather than subcortical areas seemed to be related to NSS motor signs. The latter was confirmed by Hirjak et al. (63) with a special focus on cortical morphology. The group extended their studies to the cerebellum and detected a relationship between activities of lobule VI and motor coordination as well as hard signs (102). Contrary to their previous findings, Hirjak et al. (103) showed that in addition to the cerebellum, the brainstem and basal ganglia play a role in motor regulation. Moreover, the visuospatial control of motor acts along with the sensorimotor control, as described by the above researchers (63, 101), bridges motor and sensory activation—thus supporting Dazzan's introduction of “integrative signs.” The group (104) even suggested a meaningful integration in terms of a dimensional or brain network approach as being more appropriate to understand motor abnormalities. Thus, they built on Andreasen's cortico-cerebellar-thalamo-cortical circuit (CCTCC) (64), a hypothetical large network which has influenced schizophrenia research during several decades. Hirjak et al. (104) supposed that motor function or dysfunction is related to networks which encompass the cerebello-thalamo-cortical, basal ganglia and cortico-motor circuits, as well as serotonergic in addition to dopaminergic neurotransmitters. Correspondingly, the degeneration of the indicated neurotransmitter systems may contribute to age-related fine and gross motor declines, as well as to higher cognitive deficits along with peripheral factors such as a decrease in muscle strength and slowing of movements (105).

Ad 3: Along the lines of considering CCTCC as reflecting motor as well as cognitive functioning, neurocognitive testing was included into our study. With respect to the cognitive functions tested, a parallel worsening of NSS and processing speed and/or visual search function and/or concentration was noted in our study; namely, significant correlations arose for NSS and d2 as well as NSS and TMT-A. The correlation for NSS and TMT-B was nearly significant. Processing speed may represent the most important factor among these correlations, as concentration, more so than visual search function, is a function of processing speed (106). Physiologic, functional, and structural mechanism changes lead to the cognitive decline which is present to a certain degree in all aging humans. As stated by Hirjak et al. (102), sensorimotor control, assessed via NSS, and spatial processing are both localizable to lobule VI of the cerebellum in young healthy adults; they are thus part of the CCTCC.

Another study (95) looked at the relationship between NSS and neurocognitive functioning in healthy elderly aged 60 to >80. Results of cognitive tests in this sample were in line with the literature, namely, showing a decline with age and thus supporting our findings. The same group detected moderate relations between NSS and neurocognitive parameters, especially verbal, visual, and working memories. Authors concluded that the information assessed by NSS and neurocognitive tests is nearly identical and thus may be used vice versa in the detection of cognitive decline. The question of comparability of the respective measurements had been answered in the positive in an earlier publication (42).

Although we overall agree with Chan et al. (95), we prefer not to speak of a pathological decline but of a reflection of the physiological aging process in line with Seidler et al. (105). The latter group described an age-related change of cognitive and motor systems, both of which depend on the prefrontal cortex control. In parallel to an increasing loss of WM, GM, and WBV, older individuals have to recruit a larger network to perform a task.

Cognitive change over time has been addressed exclusively in normal aging by a wide variety of researchers. It is—among others—associated with reduced WM integrity; global brain metabolism decline, which affects the left inferior frontal junction, anterior cingulate/medial prefrontal cortex, dorsomedial thalamus, and subgenual cingulate/basal forebrain (70); and atrophy of the hippocampus and prefrontal cortices (67, 68, 70, 107). Genetic factors may affect the pace and quantity of this development (68).

Taken together, results point toward an age-related increase of NSS in unison with worsening of concentration and/or visual search function as well as processing speed in healthy subjects over a lifetime. These pilot study results are in line with the literature.

### Strengths and Limitations

As studies in healthy subjects on NSS are still scarce, the main strength of this study is the census-based, homogeneous sample of healthy adults from age 20 onwards which provides a first step toward the establishment of NSS norms. Results are comparable internationally because the different scales for the assessment of NSS which are in use exhibit good psychometric properties and they comprise the same or similar items with respect to motor sequencing, motor coordination, and sensory integration. Moreover, the literature does not mention differences with respect to ethnic groups (25, 26).

It has to be borne in mind that the assessment of NSS can be learned easily by medical personnel or others. Examinations for assessment, confirmation, and follow-up of any deviations from the normal can thus be detected without technical equipment.

In our study, there was no relationship between NSS and IQ. This finding is in line with Dazzan et al. (99) who studied healthy subjects up to the age of 53. In contrast, previous researchers reported a low prevalence of NSS being related to a relatively high IQ (34, 45, 57, 108, 109). The use of different instruments may partially explain the different results and thus hint to a bias. In our study using the MWT-B, we measured crystallized and not fluid intelligence.

Certain limitations have to be mentioned, above all the cross-sectional design and the small sample size. A longitudinal design would have been the most adequate to assess NSS in healthy adults from age 20 to at least age 70. However, it is very difficult to allocate adequate resources to follow a large group of individuals for four or five decades. The small sample size does not allow to generalize findings to the general population. As there are no standard values for NSS, our preliminary data are nevertheless worthwhile. Larger studies with healthy participants are warranted. Another shortcoming is to not have asked for severe mental illness in first degree relatives. In addition, future studies may cover NSS and neurocognitive functions.

## Data Availability Statement

The datasets, i.e. raw data, presented in this article are not readily available because participating individuals were granted confidentiality. Requests to access the datasets should be directed to the first author.

## Ethics Statement

Ethical review and approval was not required for this study on healthy human participants in accordance with the local legislation and institutional requirements. The participants provided their written informed consent.

## Author Contributions

SB had the idea for this project and supervised the study and wrote the manuscript. FH drafted the study protocol. Recruitment and examination of subjects were performed by FH and MB. D-HT performed the analyses and interpretation of the data as well as a critical revision of the manuscript. All authors contributed to the article and approved the submitted version.

## Conflict of Interest

The authors declare that the research was conducted in the absence of any commercial or financial relationships that could be construed as a potential conflict of interest.
