# Supplementary material for: Neurological Soft Signs (NSS) in Census-Based, Decade-Adjusted Healthy Adults, 20 to >70 Years of Age
Source: Front Psychiatry. 2021 Jun 24;12:670539. doi: 10.3389/fpsyt.2021.670539 (PMC8264425; doi:10.3389/fpsyt.2021.670539)
Supplement: Supplementary file 1 [file Data_Sheet_1.PDF]

Tables S1-S10. Kruskal-Wallis equality-of-populations rank tests and post-hoc Dunn's pairwise comparisons

No significant differences (Kruskal-Wallis *p*-value ≥ 0.05) arose between age groups with respect to the following NSS: 3, 4, 6-9, 11, 13-15.

For the rest of NSS, there were significant differences between at least one pair of groups (Kruskal-Wallis *p*-value <0.05). Table S1 to S10 indicate which pairs of age groups were significantly from each other groups by using Dunn's test.

Table S1. Comparisons of **NSS total score** in different age groups

		NSS total		Dunn's test *p*-value					
age group	n	median	*range*	*20+*	*30+*	*40+*	*50+*	*60+*	*70+*
20+	20	6.7	2.72		0.266	0.363	0.084	**<0.001**	**<0.001**
30+	20	6.1	3.47			0.164	0.022	**<0.001**	**<0.001**
40+	20	7.3	3.76				0.152	**<0.001**	**<0.001**
50+	20	8.6	2.76					**<0.001**	**<0.001**
60+	20	15.5	4.95						0.448
70+	20	16.0	4.94						

Table S2. Comparisons of **NSS 1 / gait** in different age groups

		NSS 1		*Dunn's test p-value*					
age group	n	median	*range*	*20+*	*30+*	*40+*	*50+*	*60+*	*70+*
20+	20	0	0		0.500	0.500	0.500	0.500	**<0.001**
30+	20	0	0			0.500	0.500	0.500	**<0.001**
40+	20	0	0				0.500	0.500	**<0.001**
50+	20	0	0					0.500	**<0.001**
60+	20	0	0						**<0.001**
70+	20	0.5	0.6						

Table S3. Comparisons of **NSS 2 / tandem gait** in different age groups

		NSS 2		Dunn's test *p*-value					

age group	n	median	*range*	*20+*	*30+*	*40+*	*50+*	*60+*	*70+*
20+	20	0.30	0.57		0.399	0.268	0.033	0.005	**<0.001**
30+	20	0.35	0.59			0.359	0.056	0.009	**<0.001**
40+	20	0.40	0.50				0.110	0.023	**<0.001**
50+	20	0.70	0.73					0.221	0.003
60+	20	0.85	0.67						0.024
70+	20	1.40	0.75						

Table S4. Comparisons of **NSS 5 / Ozeretzki's test** in different age groups

		NSS 5		Dunn's test *p*-value					
age group	n	median	*range*	*20+*	*30+*	*40+*	*50+*	*60+*	*70+*
20+	20	0.25	0.44		0.438	0.070	0.037	**<0.001**	**<0.001**
30+	20	0.30	0.57			0.094	0.051	**<0.001**	**<0.001**
40+	20	0.60	0.68				0.377	0.002	0.007
50+	20	0.70	0.80					0.005	0.002
60+	20	1.35	0.75						0.377
70+	20	1.45	0.76						

Table S5. Comparisons of **NSS 10r / finger-thumb-opposition r** in different age groups

		NSS 10r		Dunn's test *p*-value					
age group	n	median	*range*	*20+*	*30+*	*40+*	*50+*	*60+*	*70+*
20+	20	0.35	0.59		0.288	0.380	0.450	**<0.001**	0.020
30+	20	0.25	0.55			0.390	0.332	0.001	0.005
40+	20	0.3	0.57				0.439	**<0.001**	0.010
50+	20	0.3	0.47					**<0.001**	0.015
60+	20	1.05	0.76						0.138
70+	20	0.8	0.77						

Table S6. Comparisons of **NSS 10l / finger-thumb-opposition l** in different age groups

		NSS 10 l		Dunn's test *p*-value					
age group	n	median	*range*	*20+*	*30+*	*40+*	*50+*	*60+*	*70+*
20+	20	0.35	0.67		0.438	0.448	0.490	**<0.001**	0.011
30+	20	0.35	0.59			0.387	0.448	0.002	0.017
40+	20	0.30	0.57				0.438	**<0.001**	0.008
50+	20	0.30	0.47					0.001	0.012
60+	20	0.95	0.69						0.203
70+	20	0.75	0.64						

Table S7. Comparisons of **NSS 12r / 2-point-discrimination r** in different age groups

		NSS 12r		Dunn's test *p*-value					
age group	n	median	*range*	*20+*	*30+*	*40+*	*50+*	*60+*	*70+*
20+	20	0	0		0.500	0.127	0.127	**<0.001**	**<0.001**
30+	20	0	0			0.127	0.127	**<0.001**	**<0.001**
40+	20	0.15	0.37				0.500	0.003	0.009
50+	20	0.15	0.37					0.030	0.009
60+	20	0.60	0.75						0.349
70+	20	0.50	0.61						

Table S8. Comparisons of **NSS 12l / 2-point-discriminiation l** in different age groups

		NSS 12l		Dunn's test *p*-value					
Age group	n	median	range	*20+*	*30+*	*40+*	*50+*	*60+*	*70+*
20+	20	0.05	0.22		0.354	0.227	0.492	**<0.001**	**<0.001**
30+	20	0	0			0.131	0.349	**<0.001**	**<0.001**
40+	20	0.15	0.37				0.236	0.006	0.003
50+	20	0.05	0.23					**<0.001**	**<0.001**

60+	20	0.65	0.88						0.389
70+	20	0.60	0.68						

Table S9. Comparisons of **NSS 16r / fist-edge-palm-test r** in different age groups

		NSS 16r		Dunn's test *p*-value					
age group	n	median	range	*20+*	*30+*	*40+*	*50+*	*60+*	*70+*
20+	20	0.30	0.57		0.337	0.449	0.279	**<0.001**	0.015
30+	20	0.20	0.41			0.385	0.157	**<0.001**	0.005
40+	20	0.25	0.44				0.237	**<0.001**	0.010
50+	20	0.40	0.60					0.001	0.055
60+	20	1.00	0.65						0.076
70+	20	0.70	0.66						

Table S10. Comparisons of **NSS 16l / fist-edge-palm-test l** in different age groups

		NSS 16l		Dunn's test *p*-value					
age group	n	median	range	*20+*	*30+*	*40+*	*50+*	*60+*	*70+*
20+	20	0.25	0.55		0.334	0.227	0.458	**<0.001**	0.034
30+	20	0.15	0.37			0.374	0.374	**<0.001**	0.012
40+	20	0.10	0.31				0.260	**<0.001**	0.005
50+	20	0.20	0.41					**<0.001**	0.027
60+	20	0.95	0.69						0.021
70+	20	0.50	0.51						
